# Labrador retrievers under primary veterinary care in the UK: demography, mortality and disorders

**DOI:** 10.1186/s40575-018-0064-x

**Published:** 2018-10-22

**Authors:** Paul D. McGreevy, Bethany J. Wilson, Caroline S. Mansfield, Dave C. Brodbelt, David B. Church, Navneet Dhand, Ricardo J. Soares Magalhães, Dan G. O’Neill

**Affiliations:** 1 Sydney School of Veterinary Science, The University of Sydney, Sydney, NSW 2006 Australia; 2 Faculty of Veterinary and Agricultural Sciences, University of Melbourne, Werribee, VIC 3030 Australia; 3 Pathobiology and Population Sciences, The Royal Veterinary College, Hawkshead Lane, North Mymms, Hatfield, Herts AL9 7TA UK; 4 Clinical Sciences and Services, The Royal Veterinary College, Hawkshead Lane, North Mymms, Hatfield, Herts AL9 7TA UK; 5 Faculty of Science, The University of Sydney, Sydney, NSW 2006 Australia; 6 UQ Spatial Epidemiology Laboratory, School of Veterinary Science, The University of Queensland, Gatton, QLD 4343 Australia; 7 Children’s Health and Environment Program, Child Health Research Centre, The University of Queensland, Brisbane, Australia

**Keywords:** VetCompass™, Electronic patient record, Breed, Pedigree, Purebred, Dog, Epidemiology, Primary-care

## Abstract

**Background:**

Labrador retrievers are reportedly predisposed to many disorders but accurate prevalence information relating to the general population are lacking. This study aimed to describe demography, mortality and commonly recorded diseases in Labrador retrievers under UK veterinary care.

**Methods:**

The VetCompass™ programme collects electronic patient record data on dogs attending UK primary-care veterinary practices. Demographic analysis covered all33,320 Labrador retrievers in the VetCompass™ database under veterinary care during 2013 while disorder and mortality data were extracted from a random sample of 2074 (6.2%) of these dogs.

**Results:**

Of the Labrador retrievers with information available, 15,427 (46.4%) were female and 15,252 (53.6%) were male. Females were more likely to be neutered than males (59.7% versus 54.8%, *P* <  0.001). The overall mean adult bodyweight was 33.0 kg (SD 6.1). Adult males were heavier (35.2 kg, SD 5.9 kg) than adult females (30.4 kg, SD 5.2 kg) (*P* <  0.001). The median longevity of Labrador retrievers overall was 12.0 years (IQR 9.9–13.8, range 0.0–16.0). The most common recorded colours were black (44.6%), yellow (27.8%) and liver/chocolate (reported from hereon as chocolate) (23.8%). The median longevity of non-chocolate coloured dogs (*n* = 139, 12.1 years, IQR 10.2–13.9, range 0.0–16.0) was longer than for chocolate coloured animals (*n* = 34, 10.7 years, IQR 9.0–12.4, range 3.8–15.5) (*P* = 0.028).

Of a random sample of 2074 (6.2%) Labrador retrievers under care in 2013 that had full disorder data extracted, 1277 (61.6%) had at least one disorder recorded. The total number of dogs who died at any date during the study was 176. The most prevalent disorders recorded were otitis externa (*n* = 215, prevalence 10.4%, 95% CI: 9.1–11.8), overweight/obesity (183, 8.8%, 95% CI: 7.6–10.1) and degenerative joint disease (115, 5.5%, 95% CI: 4.6–6.6). Overweight/obesity was not statistically significantly associated with neutering in females (8.3% of entire versus 12.5% of neutered, *P* = 0.065) but was associated with neutering in males (4.1% of entire versus 11.4% of neutered, *P* < 0.001). The prevalence of otitis externa in black dogs was 12.8%, in yellow dogs it was 17.0% but, in chocolate dogs, it rose to 23.4% (P < 0.001). Similarly, the prevalence of pyo-traumatic dermatitis in black dogs was 1.1%, in yellow dogs it was 1.6% but in chocolate dogs it rose to 4.0% (*P* = 0.011).

**Conclusions:**

The current study assists prioritisation of health issues within Labrador retrievers. The most common disorders were overweight/obesity, otitis externa and degenerative joint disease. Males were significantly heavier females. These results can alert prospective owners to potential health issues and inform breed-specific wellness checks.

## Plain English summary

With origins in the game hunting fields of Canada and developed in the UK from the 1830s, the Labrador retriever is now firmly established as one of the most globally popular dog breeds and a leading family dog. Indeed, they were the most commonly registered UK pedigree dog breed in 2016–2017. The Kennel Club currently registers three colourings: black, chocolate, or yellow [ranging from pale yellow (nearly white) to fox red]. Labrador retrievers are reportedly predisposed to many disorders but accurate prevalence information relating to the general population is lacking. This study aimed to describe demography, mortality and commonly recorded diseases in Labrador retrievers under UK veterinary care during 2013.

Clinical health records were explored for 33,320 Labrador retrievers in the VetCompass™ database under veterinary care during 2013. Of 33,320 Labrador retrievers under care in 2013, the females were more likely to be neutered than males. The most common recorded colours were black (44.6%), yellow (27.8%) and liver/chocolate (23.8%). The average adult bodyweight was 33 kg. Males were significantly heavier than females.

The median life-span of Labrador retrievers overall was 12 years but was much shorter in chocolate dogs. The most common causes of death were musculoskeletal disorders and cancer. More generally, the most common disorders affecting Labrador retrievers were overweight/obesity, ear and joint conditions. Skin and ear disease were significantly more common in chocolate dogs than in black or yellow dogs.

This report can help breeders and veterinarians prioritise strategic approaches to tackle health issues in Labrador retrievers. The results can alert prospective owners to potential health issues and inform breed-specific wellness checks.

## Background

With origins in the game hunting fields of Canada and developed in the UK from the 1830s [1], the Labrador retriever is now firmly established as one of the most globally popular dog breeds and a leading family dog. Labrador retrievers are currently very popular in the UK and were the most commonly registered UK pedigree dog breed in 2015–2016 [2]. The Kennel Club currently registers three colourings: black, liver/chocolate, or yellow (ranging from pale yellow (nearly white) to fox red [3]). We were interested in whether these pigmentations were associated with clinical disorders especially skin disease since colour is an attribute of the integument.

The median longevity of Labrador retrievers in the UK has previously been estimated at 12.5 years [4] but there s a need for additional breed-specific information on the common causes of death and any sex or coat-colour differences in longevity.

Labrador retrievers have reported predispositions to 67 diseases [5]. They are often of stocky build with a tendency to eat beyond their physiological needs, perhaps because of a pro-opiomelanocortin gene deletion [6], and can therefore be prone to obesity [7], a trait that contributes to clinical manifestations of orthopaedic problems, notably elbow and hip dysplasia [8]. Descended from dogs that were selectively bred to help fishermen retrieve nets and lost lines [9] and then bred to retrieve fallen water-fowl and other game, the breed is known for engaging in swimming. This is important because regular swimming may increase the risk of otitis externa [10] and, unless the dogs are well-dried, may lead to increased humidity in the hair-coat that may increase the prevalence of skin disorders.

A study that compared the common disorders recorded in Labrador retrievers (*n* = 339) with crossbreds (*n* = 797) attending primary veterinary practices in England suggested that Labrador retrievers are relatively predisposed to various disorders: gastrointestinal disorders (22.7% versus 18.3% in crossbreds); dermatological disorders (16.8% versus 11.9%); musculoskeletal disorders (16.2% versus 14.1%); neoplastic disorders (14.8% versus 9.2%) and obesity (12.98% versus 3.9% %) [11]. Labrador retrievers are reported in referral caseloads of veterinary dermatologists as having a predisposition to otitis externa [12].

Degenerative joint disease (DJD, often also labelled osteoarthritis), is the most common joint disease recorded in veterinary practice, and Labrador retrievers are among the breeds thought to be predisposed [13]. Specifically, by reducing mobility and therefore inducing decreased exercise and obesity, DJD has considerable potential to compromise quality of life [14]. As a degenerative condition, DJD is linked to accumulated lifetime wear-and-tear and therefore is often diagnosed in older members of breeds predisposed to obesity, especially those that have been neutered [15]. Therefore, exploration of DJD was considered of particular importance for the current study.

The VetCompass™ programme collects electronic patient record [EPR] data on dogs attending UK primary-care veterinary practices [16]. Using clinical data from the VetCompass™ programme, this study aimed to characterise the demography, longevity and common disorders of Labrador retrievers under primary veterinary care in the UK during 2013. The study was designed to build on the earlier pilot study of 418 dogs [4]. The results from the current study could provide a reliable framework to assist reforms in breeding practices and ultimately contribute to improved health and welfare of Labrador retrievers. The study was also designed to explore sex and colour associations with longevity and the prevalence of common disorders. We hypothesised that degenerative joint disease (DJD) is more prevalent in males than in females. This was predicted because males are bigger simply by having a larger skeleton and may be more predisposed to obesity [17]. We can use the results of the current study to begin to unpick these and other contributing influences on DJD.

## Methods

### Demography

Dogs recorded as Labrador retriever breed were categorised as Labrador retriever and all remaining dogs were categorised as non-Labrador retriever. The study population included all dogs under primary veterinary care at clinics participating in the VetCompass™ Programme during 2013. The VetCompass™ programme collates de-identified EPR data from collaborating practices [16]. Data fields available for analysis included a unique animal identifier from each practice management system provider along with species, breed, date of birth, sex, neuter status and bodyweight, and clinical information from free-form text clinical notes, summary diagnosis terms (VeNom codes [18]) and treatment with relevant dates.

### Body weight curves

All available bodyweight data with their associated dates were extracted from VetCompass™ database for all study Labrador retrievers (*n* = 33,320) at any date. The age at weighing (years) was calculated from the date of birth and the date of weighing. Individual bodyweight growth curves were generated for males and females by plotting age-specific bodyweights and were overlaid with a cross medians line plot using the Stata *mband* command.

All-age Bodyweight (Kg) described all available bodyweight and date combinations from the full cohort of 33,320 Labrador retrievers. Adult Bodyweight (Kg) described the mean bodyweight recorded from all body weight measurements of dogs aged over 18 months and was categorised into 5 groups (< 25 kg, 25.0–29.9 kg, 30.0–34.9 kg, 35.0–39.9 kg, ≥ 40.0 kg). Neuter described the status of the dog (entire or neutered) at the final EPR. Age described the age at the final date under veterinary care during 2013 (December 31st, 2013) and was categorised into 5 groups (< 3 years, 3.0 to < 6 years, 6.0 to < 9.0 years, 9.0 to < 12 years, ≥ 12 years).

### Longevity and cause-specific mortality

Mortality data (recorded cause, date and method of death) were extracted on deaths from the available EPR data of a random sample of 2074 (6.2%) dogs. The date of death was used to calculate the longevity of the individual and the specific cause of death, where discernible, was categorised using VeNom codes [18].

A prevalence study design derived from the cohort clinical data of dogs under veterinary care at participating practices was used to estimate the one-year period prevalence of the most commonly diagnosed disorders [19]. Sample size calculations estimated that 1730 dogs would be needed to represent a disorder with 5.0% expected prevalence to a precision of 1.0% at a 95% confidence level from a population of 33,320 dogs [20]. In this study, dogs under veterinary care were defined as those with at least one EPR; (summary diagnosis term, free-text clinical note, treatment or bodyweight) recorded either i) during 2013 and/or ii) both before and after 2013.

### Disorder prevalence

Disorder data were extracted on deaths from the available EPR data of a random sample of 2074 (6.2%) dogs. One-year (2013) period prevalence values were reported that described the probability of diagnosis at least once during 2013. Prevalence estimates were reported overall and separately by sex and by colour.

The list of unique Labrador retriever animal identification numbers was randomly ordered and a subset was reviewed manually in detail to extract the most definitive diagnoses recorded for all disorders that existed during 2013 and to manually link this to the most appropriate VeNom term as previously described [7]. Elective (e.g. neutering) or prophylactic (e.g. vaccination) clinical events were not included. No distinction was made between pre-existing and novel disorder presentations. Disorders described within the clinical notes using presenting sign terms (e.g. ‘vomiting’ or ‘vomiting and diarrhoea’), but without a formal clinical diagnostic term being recorded, were included using the first sign listed (e.g. vomiting).

The extracted diagnosis terms were mapped to a dual hierarchy of precision for analysis: fine-level precision and grouped-level precision as previously described [7]. Briefly, fine-level precision terms described the original extracted terms at the maximal diagnostic precision recorded within the clinical notes (e.g. inflammatory bowel disease would remain as inflammatory bowel disease). Grouped-level precision terms mapped the original diagnosis terms to a general level of diagnostic precision (e.g. inflammatory bowel disease would map to gastro-intestinal).

### Statistical analysis

The data were checked for internal validity and cleaned in Excel (Microsoft Office Excel 2013, Microsoft Corp.). Internal validity checks assessed for incompatibilities in extracted data: e.g. a dog that was recorded as having died but where no date of death had originally been extracted. For all inconsistencies, the original database was revisited and corrected data extracted until there were no internal inconsistencies remained in the analytic dataset. Cleaning involved standardising the terms used in the extracted dataset: e.g. the original raw data described male sex variously as ‘male’ or ‘m’. These synonymous terms were cleaned to show a single term in the analytic dataset. Analyses were conducted using Stata Version 13 (Stata Corporation). The sex, neuter status, age and adult bodyweight for Labrador retrievers under veterinary care during 2013 were described. Annual proportional birth rates described the relative proportion of Labrador retrievers compared with all dogs that were born in each year from 2004 to 2013 from the cohort that were under veterinary care in 2013.

The 95% confidence intervals (CI) estimates were derived from standard errors based on approximation to the normal distribution for disorders with ten or more events [21] or the Wilson approximation method for disorders with fewer than ten events [22]. The chi-square test was used to compare categorical variables and the Students t-test or Mann-Whitney U test to compare continuous variables as appropriate [21]. Statistical significance was set at the 5% level.

## Results

### Demography

**Fig. 1:**
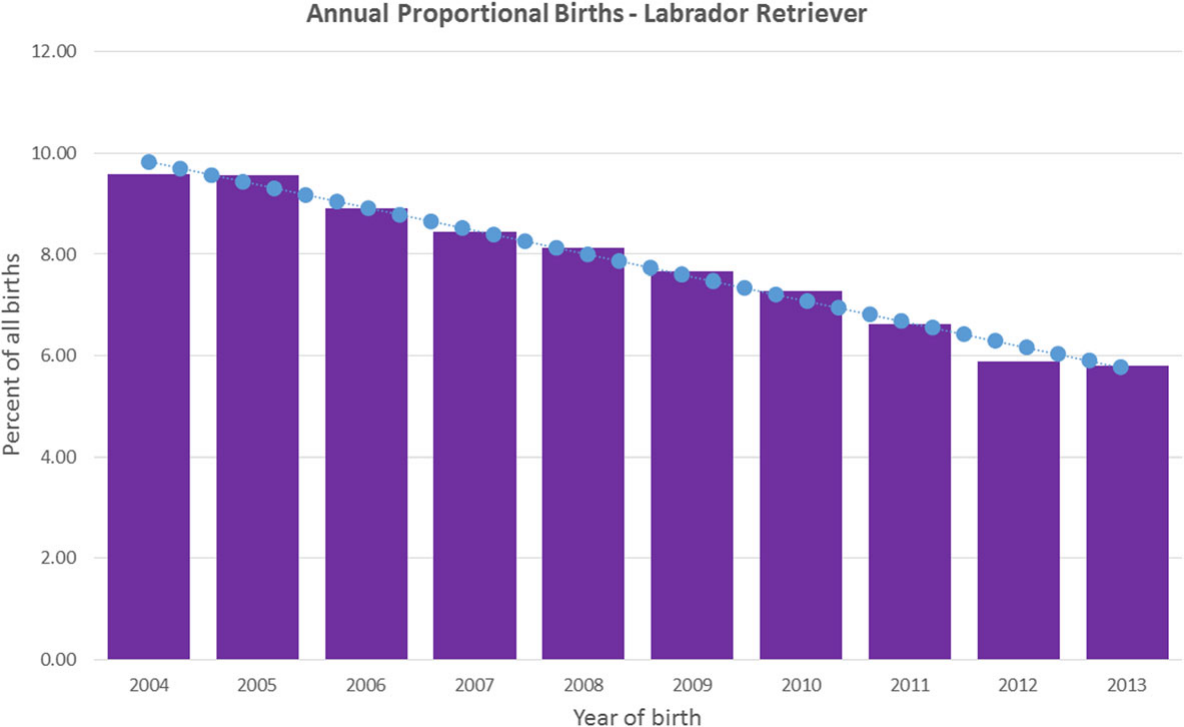
Annual proportional birth rates (2004–2013) for Labrador retrievers (*n* = 33,320) among all dogs (*n* = 455,557) attending UK primary-care veterinary clinics participating in the VetCompass™ Programme

The study population of 455,557 dogs from 304 clinics in the VetCompass™ database under primary veterinary care during 2013 included 33,320 (7.31%) Labrador retrievers. Annual proportional birth rates showed that Labrador retrievers dropped from 9.6% of the annual VetCompass™ birth cohort in 2004 to 5.8% in 2013 (Fig. 1). The most common recorded colours were black (44.6%), yellow (27.8%) and liver/chocolate (23.8%). Colour was not recorded in some dogs (*n* = 139). However, among those for which colour was recorded, the most common colours were black (44.6%), yellow (27.8%) and liver/chocolate (23.8%).

Of the Labrador retrievers with information available, 15,427 (46.4%) were female and 15,252 (53.6%) were male. Females were more likely to be neutered than males (59.7% versus 54.8%, *P* < 0.001). Data completeness varied across the variables assessed: age 99.1%, sex 99.7%, neuter 80.4% and all-age bodyweight 67.0%. The median age of the Labrador retrievers overall was 4.9 years (IQR 2.3–8.3, range 0.0–19.8) (Table 1).

**Table 1:** Demography of Labrador retrievers under primary veterinary care at practices participating in the VetCompass™ Programme in the UK from January 1st 2013 to December 31st 2013 (*n* = 33,320)

Variable	Category	Count^a^	Percent
Sex	Female	15,427	46.4
	Male	17,796	53.6
Female neuter	Entire	5007	40.3
	Neutered	7419	59.7
Male neuter	Entire	6460	45.2
	Neutered	7828	54.8
Age (years)	< 3.0	4892	32.1
	3.0–5.9	4094	26.8
	6.0–8.9	3141	20.6
	9.0–11.9	2026	13.3
	≥ 12.0	1103	7.2

^a^Count covers dogs with available data

### Body weight curves

**Fig. 2:**
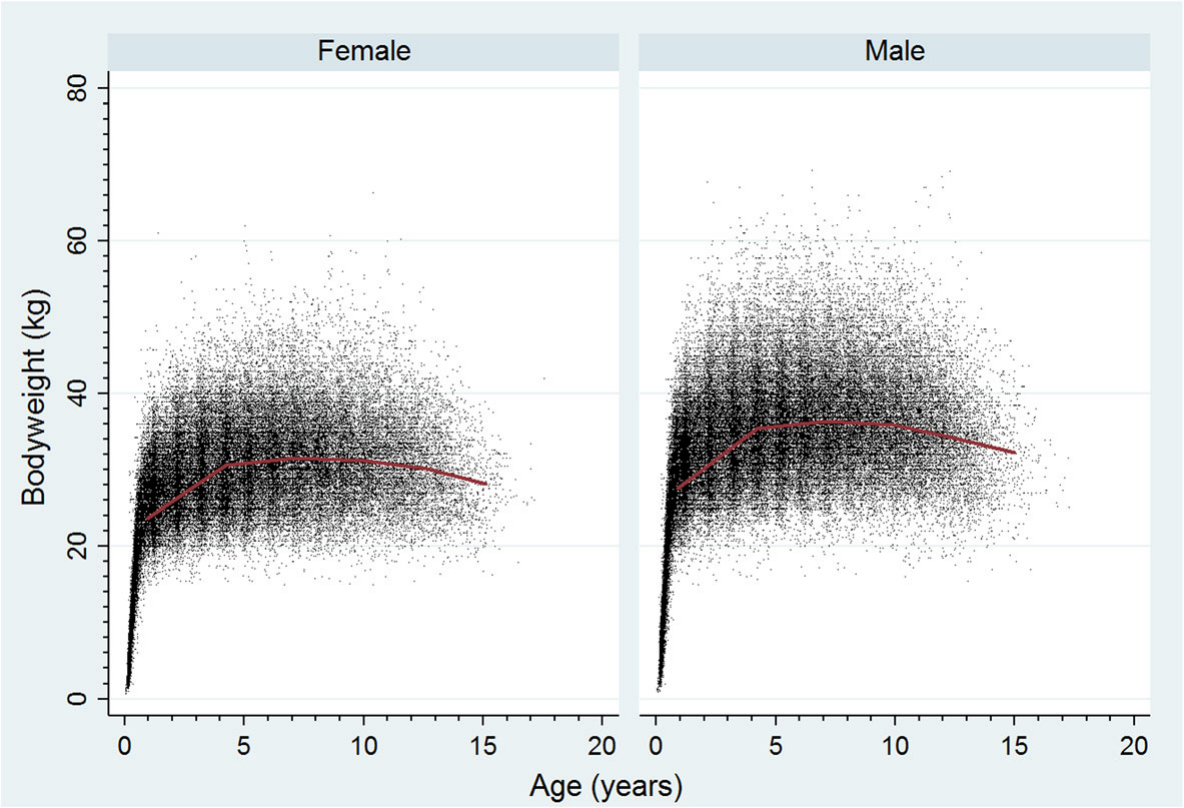
Bodyweight growth curves overlaid with a cross medians line plot for female and male Labrador retrievers attending UK primary-care veterinary clinics participating in the VetCompass™ Programme. (Females *n* = 10,228, Males *n* = 12,069)

The mean adult bodyweight overall was 33.0 kg (standard deviation [SD] 6.1). The mean adult bodyweight of males (35.2 kg, SD 5.9 kg) was higher than for females (30.4 kg, SD 5.2 kg) (*P* < 0.001). The median bodyweight across all ages for males (33.1 kg, IQR: 27.6–38.0, range: 0.9–69.3) was higher than for females was (28.7 kg, IQR: 23.9–33.0, range: 0.7–66.3) (*P* < 0.001). Bodyweight growth curves based on 84,750 bodyweight values from 10,228 females and 103,819 bodyweight values from 12,069 males showed that Labrador retriever puppies grow rapidly during their first year but that males plateau at a higher adult bodyweight than females (Fig. 2).

### Longevity and cause-specific mortality

There were 176 deaths recorded at any time during the available clinical records. The median longevity of Labrador retrievers overall was 12.0 years (IQR 9.9–13.8, range 0.0–16.0). Of 176 dogs with sex information available, the median longevity of females (12.1 years, IQR 9.5–13.8, range 00.4–16.0, *n* = 81) did not differ to males (12.0 years, IQR 10.0–13.8, range 3.8–15.4, *n* = 91) (*P* = 0.856). The median longevity of neutered animals (12.5 years, IQR 10.5–13.9, range 5.5–16.0) was longer than for entire animals (11.6 years, IQR 8.9–12.4, range 0.0–15.2) (*P* = 0.010). There were 29 (16.5%) deaths that did not have a cause of death stated. Of the remaining 147 deaths, the most common causes of death described at a grouped-precision level were musculoskeletal disorder (*n* = 36, prevalence 24.5%) and neoplasia (31, 21.1%). The probability of death did not differ between males and females for any of the 10 most common causes of mortality (Table 2). The median age at death from these 10 causes varied from 9.1 years (IQR: 8.2–12.1 years) for heart disease to 13.4 years (interquartile range: 11.5–14.0 years) for musculoskeletal disorders (Table 2). The median longevity of non-chocolate coloured dogs (*n* = 139, 12.1 years, IQR 10.2–13.9, range 0.0–16.0) was longer than for chocolate coloured animals (*n* = 34, 10.7 years, IQR 9.0–12.4, range 3.8–15.5) (*P* = 0.028).

**Table 2:** Mortality in Labrador retrievers with a recorded cause of death under primary-care veterinary at UK practices participating in the VetCompass™ Programme from January 1st, 2013 to December 31st, 2013 (*n* = 147)

Grouped-level disorder	Overall Count (%)	Female count (%)	Male Count (%)	*P*-Value male vs female	Age at death [years]: Median (interquartile range)
Musculoskeletal disorder	36 (24.5)	18 (24.3)	18 (25.0)	0.925	13.4 (11.5–14.0)
Neoplasia	31 (21.1)	13 (17.6)	18 (25.0)	0.272	10.6 (8.5–12.4)
Mass lesion	11 (7.5)	5 (6.8)	6 (8.3)	0.718	9.7 (9.0–10.1)
Brain disorder	8 (5.4)	5 (6.8)	3 (4.2)	0.492	11.8 (9.5–13.1)
Liver disorder	8 (5.4)	3 (4.1)	5 (6.9)	0.443	12.0 (9.3–12.9)
Renal disease	8 (5.4)	5 (6.8)	3 (4.2)	0.492	11.3 (8.7–13.3)
Endocrine disorder	7 (4.8)	5 (6.8)	2 (2.8)	0.261	12.1 (11.1–13.7)
Enteropathy	5 (3.4)	3 (4.1)	2 (2.8)	0.672	10.3 (5.4–11.0)
Heart disease	5 (3.4)	2 (2.7)	3 (4.2)	0.627	9.1 (8.2–12.1)
Urinary system disorder	4 (2.7)	1 (1.4)	3 (4.2)	0.297	12.7 (11.5–13.9)
Other	24 (16.3)				
Total	147 (100)				

The *P*-value reflects comparison between the prevalence in females and males

### Disorder prevalence

The EPRs of a random sample of 2074 (6.2%) of Labrador retrievers were manually examined to extract all recorded disorder data for 2013. There were 1277 (61.6%) Labrador retrievers with at least one disorder recorded during 2013 while the remaining 38.4% had no disorder recorded and either presented for prophylactic management only or did not present at all during 2013. The median count of disorders per Labrador retriever during 2013 was 1 disorder (IQR 0–2, range 0–11) and did not differ between females (median 1, IQR 0–2, range 0–11) and males (median 1, IQR 0–2, range 0–7) (*P* = 0.796).

The study included 2291 unique disorder events recorded during 2013 that encompassed 254 distinct fine-level disorder terms. The most prevalent fine-level precision disorders recorded were otitis externa (*n* = 215, prevalence 10.4%, 95% CI: 9.1–11.8), overweight/obesity (183, 8.8%, 95% CI: 7.6–10.1), degenerative joint disease (115, 5.5%, 95% CI: 4.6–6.6), lameness (91, 4.4%, 05% CI: 3.5–5.4) and periodontal disease (87, 4.2%, 95% CI: 3.4–5.1). Among the 20 most common fine-level precision disorders, males were more likely than females to be diagnosed with vomiting (4.6% versus 2.5% respectively, *P* = 0.009) (Table 3). Overweight/obesity was not statistically significantly associated with neutering in females (8.3% of entire versus 12.5% of neutered, *P* = 0.065) but was associated with neutering in males (4.1% of entire versus 11.4% of neutered, *P* < 0.001). There some significant associations between on coat colour associations with ear and skin disease (see Table 5). The prevalence of otitis externa in black dogs was 12.8%, in yellow dogs it was 17.0% but, in chocolate dogs, it rose to 23.4% (P < 0.001). Similarly, the prevalence of pyo-traumatic dermatitis in black dogs was 1.1%, in yellow dogs it was 1.6% but in chocolate dogs it rose to 4.0% (*P* = 0.011).

**Table 3:** Prevalence of the most common disorders at a fine-level of diagnostic precision recorded in Labrador retrievers (*n* = 2074) attending UK primary-care veterinary practices participating in the VetCompass™ Programme from January 1st, 2013 to December 31st, 2013

Fine-level disorder	Count	Overall prevalence %	95% CI	Female prevalence %	Male prevalence %	*P*-Value
Otitis externa	215	10.4	9.1–11.8	10.3	10.6	0.821
Overweight/obesity	183	8.8	7.6–10.1	9.8	8.0	0.139
Degenerative joint disease	115	5.5	4.6–6.6	5.9	5.2	0.480
Lameness	91	4.4	3.5–5.4	4.1	4.7	0.521
Periodontal disease	87	4.2	3.4–5.1	4.5	3.9	0.525
Lipoma	85	4.1	3.3–5.0	3.8	4.4	0.484
Vomiting	74	3.6	2.8–4.5	2.5	4.6	**0.009**
Diarrhoea	67	3.2	2.5–4.1	2.9	3.4	0.494
Conjunctivitis	57	2.7	2.1–3.5	3.0	2.6	0.577
Skin mass	51	2.5	1.8–3.2	2.4	2.6	0.756
Pruritus	43	2.1	1.5–2.8	2.2	2.0	0.832
Anal sac impaction	38	1.8	1.3–2.5	1.6	2.0	0.522
Pyoderma	36	1.7	1.2–2.4	1.5	1.9	0.499
Coughing	33	1.6	1.1–2.2	1.6	1.6	0.885
Stiffness	33	1.6	1.1–2.2	2.2	1.1	0.057
Undesirable behaviour	31	1.5	1.0–2.1	1.3	1.7	0.551
Pyo-traumatic dermatitis	28	1.4	0.9–1.9	1.1	1.6	0.396
Alopecia	26	1.3	0.8–1.8	1.0	1.5	0.367
Pododermatitis	26	1.3	0.8–1.8	1.0	1.5	0.367
Laceration	25	1.2	0.8–1.8	1.3	1.1	0.632

The *P*-value reflects prevalence comparison between females and males

*P*-values in bold are statistically significant

*CI* confidence interval

There were 51 distinct grouped-level precision disorder terms recorded. The most prevalent grouped-level precision disorders were musculoskeletal (*n* = 261, prevalence: 12.6%, 95% CI: 11.1–14.1), aural (219, 10.6%, 95% CI: 9.3–12.0), gastrointestinal (210, 10.1%, 95% CI: 8.9–11.5), dermatological (202, 9.7%, 95% CI: 8.5–11.1) and overweight/obesity (183, 8.8%, 95% CI: 7.6–10.1). Among the 15 most common grouped-level precision disorders, females were more likely than males to be diagnosed with a urinary system disorder (2.3% versus 0.9% respectively, *P* = 0.014) (Table 4).

**Table 4:** Prevalence of the most common grouped-level disorders recorded in Labrador retrievers (*n* = 2074) attending UK primary-care veterinary practices participating in the VetCompass™ Programme from January 1st 2013 to December 31st 2013

Grouped-level disorder	Count	Overall prevalence	95% CI	Female prevalence %	Male prevalence %	*P*-Value
Musculoskeletal	261	12.6	11.1–14.1	13.5	11.8	0.248
Aural	219	10.6	9.3–12.0	10.5	10.7	0.835
Enteropathy	210	10.1	8.9–11.5	8.7	11.3	0.052
Dermatological	202	9.7	8.5–11.1	9.6	9.9	0.832
Overweight/obesity	183	8.8	7.6–10.1	9.8	8.0	0.139
Neoplasia	153	7.4	6.3–8.6	7.4	7.4	0.963
Dental	114	5.5	4.6–6.6	5.7	5.3	0.679
Mass-associated	100	4.8	3.9–5.8	4.7	5.0	0.799
Ophthalmological	97	4.7	3.8–5.7	5.3	4.0	0.164
Traumatic	73	3.5	2.8–4.4	3.0	4.0	0.190
Upper respiratory tract	54	2.6	2.0–3.4	2.7	2.6	0.892
Undesirable behaviour	53	2.6	1.9–3.3	2.4	2.8	0.570
Anal sac	46	2.2	1.6–2.9	2.1	2.4	0.605
Parasitic	36	1.7	1.2–2.4	1.6	1.8	0.735
Urinary system	32	1.5	1.1–2.2	2.3	0.9	**0.014**

The *P*-value reflects prevalence comparison between females and males

*P*-values in bold are statistically significant

*CI* confidence interval

## Discussion

The main findings from the current study are that the most common disorders among Labrador retrievers were overweight/obesity, otitis externa and degenerative joint disease. Overweight/obesity was not statistically significantly associated with neutering in females but was associated with neutering in males. This is important not least because males were significantly heavier.

### Demography and mortality

The median longevity of Labrador retrievers in the current study overall was 12.0 years; this was similar to a previous estimate, based on a sample of 418 dogs, of 12.5 years [7]. The overall median longevity for dogs of 12.0 years reported here aligns with the historic median estimate of 12.0 years among Labrador retrievers insured in the UK or attending dog shows [23]. From the current sample, the median longevity of females did not differ to males, but the median longevity of neutered animals was longer than for entire animals. This is in keeping with studies of other breeds [7].

The current study has shown that annual proportional birth rates for Labrador retrievers in the UK dropped from 9.6% of the annual VetCompass™ birth cohort in 2004 to 5.8% in 2013. By definition, this drop represents an underestimate of early cohorts because 20–25% of early cohorts are likely to have died before 2013 and thus are not in the dataset.

The apparent drop in the relative popularity of Labrador retrievers may reflect an indirect effect of the nascent rise in popularity of the brachycephalic breeds, such as French bulldogs, whose registrations rose from third in 2016 to second in 2017 [2]. The decline of the annual proportional birth rates for Labrador retrievers in the UK may also reflect the rise of designer hybrid cross-bred dogs, notably poodle crosses. Unfortunately, the true scale of the rise in the popularity of such crosses is difficult to monitor since litters are not registered. Equally, monitoring the health of these dogs is hampered by the persistent absence of verified data on parentage to establish which are first crosses, second crosses or other [24].

The relationship between coat colour and longevity is intriguing and has not, to the authors’ knowledge, been reported in other breeds. The significantly shorter lifespan of chocolate dogs compared with non-chocolate dogs may reflect differences in lifetime burden of disease, notably disorders of the integument (see below), that may create differences in accumulated immune response.

In the current study, the most common causes of death described at a grouped-precision level were musculoskeletal disorder and neoplasia. Of these two disorders, neoplasia had more effect on longevity than musculoskeletal disease, being associated with a median age at death of 10.6 years versus 13.4 years. Although, one of the important causes of death was neoplasia, cancer did not figure as being a disease of major prevalence. This serves to highlight that most common diseases are not terminal and that disease predisposition in life is not the same as disease predisposition as a cause of death.

In males, the possible benefits of neutering may include reducing the risk of later testicular disease, and reducing the risks from androgen-dependent disorders such as perineal hernias, perineal adenomas, prostatitis and benign prostatic hyperplasia [25]. In females, neutering was also reported to reduce the risk of mammary neoplasia [26], but a recent review stated that the evidence for such an association as only weak [27]. The association of neutering to longevity could be more closely associated to the level of health care provided by conscientious owners, but this has yet to be validated in any external study.

### Colour

There some significant associations between on coat colour associations with ear and skin disease (see Table 5). The prevalence of otitis externa was significantly higher in chocolate dogs than in non- chocolate dogs. Similarly, the prevalence of pyo-traumatic dermatitis was more than twice as high in chocolate dogs than in non- chocolate dogs. We were interested in the association with coat colour because chocolate pigmentation is recessive in dogs [28]. So, if chocolate coat colour is desired in litters, breeders may be motivate to breed from certain lines that may inadvertently increase the ensuing puppies’ predisposition to certain diseases. It is possible that a more restricted population gene pool has a higher carriage rate of the disease risk genes involved in ear and skin conditions. This finding merits further investigation, for example, in the extant VetCompass™ databases for other differentially pigmented breeds such as pugs and Cavalier King Charles spaniels.

**Table 5:** Colour variation among Labrador retrievers in their prevalence of the most common disorders at a fine-level of diagnostic precision recorded at UK primary-care veterinary practices participating in the VetCompass™ Programme from January 1st, 2013 to December 31st, 2013

Fine-level disorder	Overall prevalence %	Black prevalence %	Chocolate prevalence %	Yellow prevalence %	*P*-Value
Otitis externa	10.4	12.8	23.4	17.0	**< 0.001**
Overweight/obesity	8.8	13.0	15.4	16.7	0.272
Degenerative joint disease	5.5	10.7	6.7	8.8	0.152
Lameness	4.4	6.9	6.7	7.7	0.850
Periodontal disease	4.2	7.6	5.7	6.9	0.581
Lipoma	4.1	7.2	4.7	8.0	0.217
Vomiting	3.6	6.0	6.7	4.0	0.259
Diarrhoea	3.2	5.4	6.4	4.2	0.468
Conjunctivitis	2.7	4.3	5.0	4.5	0.900
Skin mass	2.5	3.8	4.7	3.5	0.701
Pruritus	2.1	2.9	4.7	3.2	0.371
Anal sac impaction	1.8	3.4	2.0	2.7	0.473
Pyoderma	1.7	2.9	4.0	2.1	0.349
Coughing	1.6	2.5	3.3	2.1	0.605
Stiffness	1.6	2.4	3.0	2.4	0.824
Undesirable behaviour	1.5	3.3	1.7	2.1	0.314
Pyo-traumatic dermatitis	1.4	1.1	4.0	1.6	**0.011**
Alopecia	1.3	2.0	3.0	1.3	0.303
Pododermatitis	1.3	1.4	3.0	1.9	0.284
Laceration	1.2	2.4	1.7	1.6	0.661

The *P*-value reflects prevalence comparison between the three colours. (*n* = 2074)

*P*-values in bold are statistically significant

### Obesity

The current results for obesity indicate that Labrador retrievers are at increased risk of being overweight or obese. If we compare the current results to the results for other VetCompass™ breed studies using the same methodology, we can see that the prevalence in Labrador retrievers of overweight/obese of 8.8% (95% CI: 7.6–10.1) is less than that reported for pugs (prevalence: 13.18%, 95% CI: 11.12–15.43 [29]) but more than that for Border terriers (7.01%, 95% CI: 5.69–8.52 [30]) Rottweilers (7.06%, 95% CI: 6.02–8.21 [31]), German shepherd dogs (5.18%, 95% CI: 4.16–6.36 [32]) and French bulldogs, for which overweight/obese did not appear in the top 25 most common disorders [33]. As with all studies of canine obesity that rely on attending veterinarians’ subjective assessments of bodyweight, the current data rely entirely on veterinarians recording this information in the text of the clinical record. Furthermore, we note that the terms obesity and overweight are often used synonymously and that, especially for breed-specific studies such as the current one, actual bodyweight is more informative than these overarching and often overlapping labels.

Excessive bodyweight (overweight and obesity) is very common in domestic dogs, and linked to various associated conditions, such as diabetes and cardiovascular disease, and also to reduced longevity [12]. Weight loss is known to improve quality of life in dogs [11, 34] reflecting other benefits such as improved insulin resistance [35] and reduced lameness [36]. Weight loss can be achieved through dietary energy restriction [37] along with increased protein:fat dietary rations, but compliance to the diet must be maintained [38]. Physical activity should be part of a weight reduction programme.

Complementary interactions of dog mobility and physical activity have been shown for both dogs and their owners [39, 40], and it has been proposed that obese owners may be more likely to have obese dogs [41–43]. Males plateau at a higher adult bodyweight than females, and neutered dogs are more prone to obesity than entire dogs [44, 45]. These associations may be due to a reduced metabolic rate [46, 47]. The current analysis did not explore the temporality of whether dogs were neutered before or after being classified as overweight; a possible avenue for future research on data from EPR. In the current Labrador retriever sample, only males were significantly predisposed to obesity when neutered. The question of when, during the maturation of Labrador retrievers, this dimorphism arises warrants deeper scrutiny. The generalised decline in bodyweight from 10 years onward may reflect an effect of wasting disorders and flux in the ratio of muscle to bone mass.

Apart from obesity/overweight (discussed above), the main disorders that merit discussion in the current report are musculoskeletal, periodontal, enteropathy, aural and urinary disease. The results relating to these conditions are explored sequentially below.

### Musculoskeletal

The hypothesis that DJD is more prevalent in males than in females was not supported by the current data. This finding is consistent with a recent report that focused on appendicular arthritis [13]. Musculoskeletal disorders, primarily identified as DJD, lameness and “stiffness” at the fine diagnostic level, proved to be the most prevalent group level disorder, with 261 counted among the 2074 in our random cohort of Labrador retrievers (See Table 3). It was also the most common recorded cause of death, accounting for nearly a quarter of all mortalities. This finding is consistent with a study of 212 insured Swedish Labradors in that 29% of mortalities were attributed to non-traumatic and non-neoplastic musculoskeletal disorders [48].

DJD accounted for at least 115 of the 261 counted cases of musculoskeletal disorders (44%) in the current study and potentially an unknown number of cases characterised as lameness or stiffness at the fine level of diagnostic precision. In dogs, DJD is usually secondary to a primary joint problem such as a traumatic injury, a developmental abnormality or, more rarely, infectious or autoimmune inflammation [49–51]. Labrador retrievers are known to be at risk for common developmental joint disorders including canine elbow dysplasia [52–54], canine hip dysplasia [52–55], and humeral head osteochondrosis [53, 56] and are also prone to cranial cruciate ligament rupture [57].

In these diseases, developmental joint incongruity leading to abnormal transmission of weight bearing forces and/or failure of endochondral ossification are believed to lead to progressive cartilage damage, and subsequent DJD [58]. Similarly, degenerative joint disease develops progressively in canine hip dysplasia due to the transmission of weight-bearing forces though abnormally loose and increasingly dysplastic hip joints [59], and humeral head osteochondrosis results from failure of endochondral ossification. While several of these conditions have sex predispositions reported in the literature in some studies [53, 54, 57, 60], cases of DJD were not significantly different by sex in this study.

The current study identified a further 91 cases of lameness and 33 cases of stiffness, in addition to the 115 cases of DJD. Diagnostic terms such as lameness and stiffness while non-specific could represent milder or less thoroughly investigated cases of DJD or acute/sub-acute primary injuries which could predispose the dog to secondary DJD such as an initial presentation of a cranial cruciate ligament rupture, traumatic injuries and fractures, neurological conditions and other miscellaneous disorders. We acknowledge the risk that including some presenting signs that are not diagnostic may compromise phenotypic rigour. For example, by regarding “stiffness” as a musculoskeletal term, we may risk including some dogs that have weakness from a systemic disorder, such as pyrexia, rather than a strictly musculoskeletal disease. Further investigation could help elucidate the extent to which “lameness” and “stiffness” are used as a euphemism for DJD.

The current results do not suggest more frequent DJD or obesity in males. We acknowledge that it is difficult to disambiguate the development of the musculoskeletal disorders and obesity. It appears that male dogs are less likely to be diagnosed as obese even when heavier perhaps, as has been proposed in various breeds of cats because of having a larger frame [61]. Also some of the causal factors of DJD have (often inconsistent) sex predispositions reported in the literature [52–54], so if males are more inclined to obesity this might be offset by a lower tendency to certain diseases which lead to secondary DJD. Future iterations of this analysis should consider a closer investigation of the determinants of the patterns of disorders identified in this study.

### Periodontal disease

Periodontal disease was a common finding in the current population of Labrador retrievers. If we compare the current results for Labrador retrievers to the results for other VetCompass™ breed studies using the same methodology, we can see that the prevalence of periodontal disease of 4.2% (95% CI: 3.4–5.1) is less than that reported for pugs (prevalence: 6.14%, 95% CI: 4.74–7.81 [29]) but much more than that for German shepherd dogs (1.14%, 95% CI: 0.69–1.78 [32]). Given that Labrador retrievers are mesocephalic, whereas pugs are brachycephalic and German shepherd dogs tend towards dolichocephalism, this suggests that cephalic index may have a bearing on periodontal health [62] and seems to merit further investigation. That said, periodontal disease had a prevalence of 17.63% in (mesocephalic) Border terriers (95% CI: 15.62–19.79 [30]) does not appear in the list of the 26 most common disorders recorded in French Bulldogs [33].

### Gastrointestinal disease

Gastrointestinal disease had an overall prevalence of 10.1% in the current sample (95% CI: 8.9–11.5). It encompasses a variety of disorders including pancreatitis, idiopathic gastroenteritis, dietary indiscretion, intestinal foreign bodies, infectious gastroenteritis and chronic conditions such as inflammatory bowel disease. There have been no studies of overall gastrointestinal disease diagnosed at veterinary practices in dogs, but a telephone survey of owners did report gastrointestinal disease as one of the major disease presentation [63]. Conversely, in another study of pedigree dogs visiting first opinion veterinary practices in the UK, gastrointestinal disease was not one of the most prevalent disorders [7]; additionally, Labrador retrievers have been reported to have a decreased risk of acute pancreatitis [64]. Potential reasons for Labrador retrievers to have a higher incidence of gastrointestinal disorders includes their anecdotal propensity to scavenge food [35] and hence potentially a higher incidence of disease such as gastroenteritis or foreign body obstruction. Although the level of detail is not adequate from our analysis to determine the true primary diagnosis for each case, the large number of clinical records evaluated is likely more reflective of the true incidence in the general population. The current finding that males are significantly more likely than females to present with vomiting merits further scrutiny but there is a need for caution here since vomiting may be reported as part of a suite of gastrointestinal disease. It is also interesting that vomiting was more prevalent than diarrhoea in the current population of Labrador retrievers [3.6% (CI: 28–4.5) versus 3.2% (CI: 2.5–4.5)] whereas the reverse trend was apparent in German shepherd dogs [29] [2.53% (CI1.83–3.40) versus 5.24% (CI: 4.22–6.42] using the same methodology and in Labrador retrievers using a longitudinal cohort design [65].

### Aural and dermatological

Given that both conditions affect the integument, we shall consider aural and dermatological diseases together. Aural disease was common among the current population of Labrador retrievers with a prevalence of 10.6% (95% CI: 9.3–12.0) but was less than that reported for pugs (prevalence: 15.06%, 95% CI: 12.91–17.42 [29]), French Bulldogs (14.0%, 95% CI: 12.6–15.5 [33]) and German shepherd dogs (11.14%, 95% CI: 9.67–12.76 [329]). Similarly, the prevalence of dermatological (or cutaneous) diseases in Labrador retrievers was considerable at 9.7% (95% CI: 8.5–11.1) and more than that reported for Rottweilers (2.91%, 95% CI: 2.25–3.70 [31]) but again less than that reported for pugs (prevalence: 15.60%, 95% CI: 13.38–17.95 [29]) and for German shepherd dogs (13.98%, 95% CI: 12.34–15.74 [33]). Hair coat length and aural conformation may influence predisposition to these disorders but most of the disorders are related to atopy. The predisposition of chocolate Labrador retrievers in the current sample suggests further avenues of immunological research within the breed.

Labrador retrievers are reported in dermatology referral caseloads as having a predisposition to otitis [9]. Dermatological problems in our cohort included atopic dermatitis (that may account for the accompanying prevalence of otitis externa) and pyo-traumatic dermatitis that may reflect to some extent the breed’s fondness for swimming and retrieving from water. Otitis externa is one of the most common problems reported in canine practice [66], as acute cases manifest with head-shaking that is distressing for dogs and owners alike, dogs are often presented swiftly and can be managed with topical polyvalent ear preparations. A range of organisms can be implicated in cases of otitis, including Gram-positive cocci, Gram-negative rods such as *Pseudomonas*, and the yeast *Malassezia pachydermatis* [67]. However, Nuttall [66] states that in most cases bacterial culture and sensitivity testing is not usually performed, and cytology can be helpful in identifying the most likely causative organisms. However, allergic disease, notably atopic dermatitis, is the most common primary trigger for otitis externa [9]. Indeed, a review of referred cases of otitis in dogs concluded that 75% had atopic dermatitis as a primary trigger [9]. Acute otitis externa cases frequently progress into chronic or recurrent disease that is much harder to resolve, and along with accumulated immunological events, are thought to increases the risk of aural haematomata [68]. Refractory cases are particularly problematic in fearful dogs that learn to avoid having their ears examined and treated by owners.

### Urinary disease

Females in the current study were more likely than males to be diagnosed with a urinary system disorder. This sex-related difference is unlikely to be peculiar to Labrador retrievers because it is reported that, apart from obstructions, urinary disorders are generally more common in females than males with, for example, urinary tract infections being more than twice as common [69]. Additionally, urinary incontinence due to urethral sphincter mechanism incontinence occurs predominantly in neutered female dogs [70] and has been reported to occur more frequently in larger-breed animals [71]. Urinary system disorders were recorded as a cause of death in only 2.7% of Labrador retrievers in the current study and specific details on the frequency of individual urinary conditions were not collated as part of the current study.

### Study limitations

The findings of this study should be interpreted in light of some limitations. First, we have considered a random sample of dogs from the VetCompass™ UK database with the objective of estimation of prevalence of common disorders, which may underpowered for less common disorders or causes of mortality. In this study, as a secondary aim, measures of disease frequency for cause-specific mortality were quantified from clinical records, which may arguably not provide an accurate representation of all mortality events in the population. In a similar vein, we acknowledge that, although VetCompass™ offers the best resource currently available for studying the national dog population in the UK, demography of an entire population, rather than the veterinary population, may be difficult to infer from clinical data. The current report includes the results of multiple testing and therefore strict adherence to a 0.05 *p*-value cut-off risks Type 1 error of accepting false positive findings. We suggest that the readers explore the differences in the reported prevalence or other results to understand the meaning of these values rather than relying on *p*-values [72]. Some of the results reported in this study were based on relatively small sample sizes and therefore the risks of Type II error (false negative) need to be considered for these analyses. The focus of the current article was on disorder prevalence rather than mortality. Future studies looking more closely at the latter outcomes could be designed could focus on all dead animals reported in the database as a starting point (with a record of death) and investigate the mortality status of those assumed alive by following up with their owners.

## Conclusion

This study of over two thousand Labrador retrievers provides important disorder information on the general population of Labrador retrievers. The most common disorders in Labrador retrievers were otitis externa, overweight/obesity and degenerative joint disease. Otitis externa and pyo-traumatic dermatitis were less prevalent in black dogs yellow dogs than in chocolate dogs. Chocolate dogs had a significantly shorter lifespan than non-chocolate dogs. These results provide a framework to identify health priorities in Labrador retrievers and can contribute positively to reforms to improve health and welfare within the breed.

## Abbreviations

CI: Confidence interval
DJD: Degenerative joint disease
EPR: Electronic patient record
IQR: Interquartile range
SD: Standard deviation
